# Oocyte and embryo developmental competence following small multiple cyclophosphamide dose administrations in prepubertal female mice are comparable to adolescents

**DOI:** 10.1038/s41598-025-33355-2

**Published:** 2025-12-29

**Authors:** Sujith Raj Salian, Dhakshanya Predheepan, Akshatha Daddangadi, Vani R Lakshmi, Sindhura Lakshmi Koulmane Laxminarayana, Guruprasad Kalthur, Shubhashree Uppangala, Satish Kumar Adiga

**Affiliations:** 1 Centre of Excellence in Clinical Embryology, Kasturba Medical College, Manipal, Manipal Academy of Higher Education, Manipal, 576104 India; 2 Centre for Fertility Preservation, Department of Reproductive Science, Kasturba Medical College, Manipal, Manipal Academy of Higher Education, Manipal, 576104 India; 3 Department of Health Technology and Informatics, Prasanna School of Public Health, Manipal Academy of Higher Education, Manipal, Karnataka India; 4 Department of Pathology, Kasturba Medical College, Manipal, Manipal Academy of Higher Education, Manipal, 576104 India; 5 Division of Reproductive Biology, Department of Reproductive Science, Kasturba Medical College, Manipal, Manipal Academy of Higher Education, Manipal, 576104 India; 6 Division of Reproductive Genetics, Department of Reproductive Science, Kasturba Medical College, Manipal, Manipal Academy of Higher Education, Manipal, 576104 India

**Keywords:** Cyclophosphamide, Long-term side effects, Ovarian follicular pool, Embryo development, Blastocyst quality, Cancer, Developmental biology

## Abstract

A small multiple-dose regimen of cyclophosphamide (CY) compared to a large single dose in prepubertal mice has shown reduced follicle loss and improved fertility outcomes at early reproductive age. However, the long-term effects on the competency of oocytes from such surviving follicles, compared to those at post-pubertal age, remain unknown. Small multiple CY (75 mg/Kg, four weekly doses) injections were administered to prepubertal (PP75X4) and adolescent (AD75X4) Swiss albino female mice. At 22 weeks of age, surviving females were assessed for follicular pool and oocyte functional competency. The resulting embryos were assessed for blastulation, total cell number (TCN) quality, and in vitro inner cell mass (ICM) progression. At 22 weeks of life, PP75X4 females had a three-fold reduction (*p* < 0.001) in the total number of follicles compared to the untreated control. However, the differences between PP75X4 and AD75X4 were not statistically significant. Though PP75X4 females demonstrated a significant (*p* < 0.001) reduction in oocyte yield when compared to AD75X4, embryo quality, blastulation, TCN, and apoptosis were comparable between PP75X4 and AD75X4 groups. Small multiple doses of CY lead to reduced oocyte yield in prepubertal mice compared to adolescents. This study suggests that oocytes from prepubertal and adolescent individuals receiving small multiple doses of CY exhibit comparable functional competence, resulting in embryos of similar quality.

**Supplementary Information:**

The online version contains supplementary material available at 10.1038/s41598-025-33355-2.

## Introduction

Cyclophosphamide (CY) is a commonly used chemotherapeutic agent in oncology to treat cancer conditions and certain autoimmune disorders across all age groups^1^. However, CY exposure to ovaries has been demonstrated to be toxic as CY accelerates the activation of follicles by PI3K/AKT/FOXO3a signaling pathway^2^ or the depletion of follicles by p53 activation-mediated apoptosis^3^, which raises fertility concerns in cancer survivors^4–6^. Importantly, reports on the adverse effects of CY on murine ovaries have demonstrated to be dose-dependent^7^. A recent study revealed that a small multiple CY dose regimen (75 mg/Kg, four times weekly) in prepubertal mice showed reduced follicle loss^3^ and improved fertility outcomes during early reproductive age compared to a single large dose^8^. Nevertheless, the fate of the surviving follicles, the long-term consequences, and the age-dependent response of the small multiple CY dose regimen remain a concern^9,10^. Importantly, while CY exposure in adult mice has been shown to cause abnormalities in oocytes and granulosa cells in the surviving follicles, the long-lasting consequence of CY exposure in prepubertal mice is underexplored^9^. Therefore, here we made an attempt to investigate the functional ability of oocytes and preimplantation embryos following exposure to a small multiple CY dose regimen (75 mg/Kg, four weekly doses) at prepubertal (PP75X4) and adolescent (AD75X4) age.

## Results

### Ovarian follicular pool and oocyte yield in PP75X4 and AD75X4

**Fig. 1:**
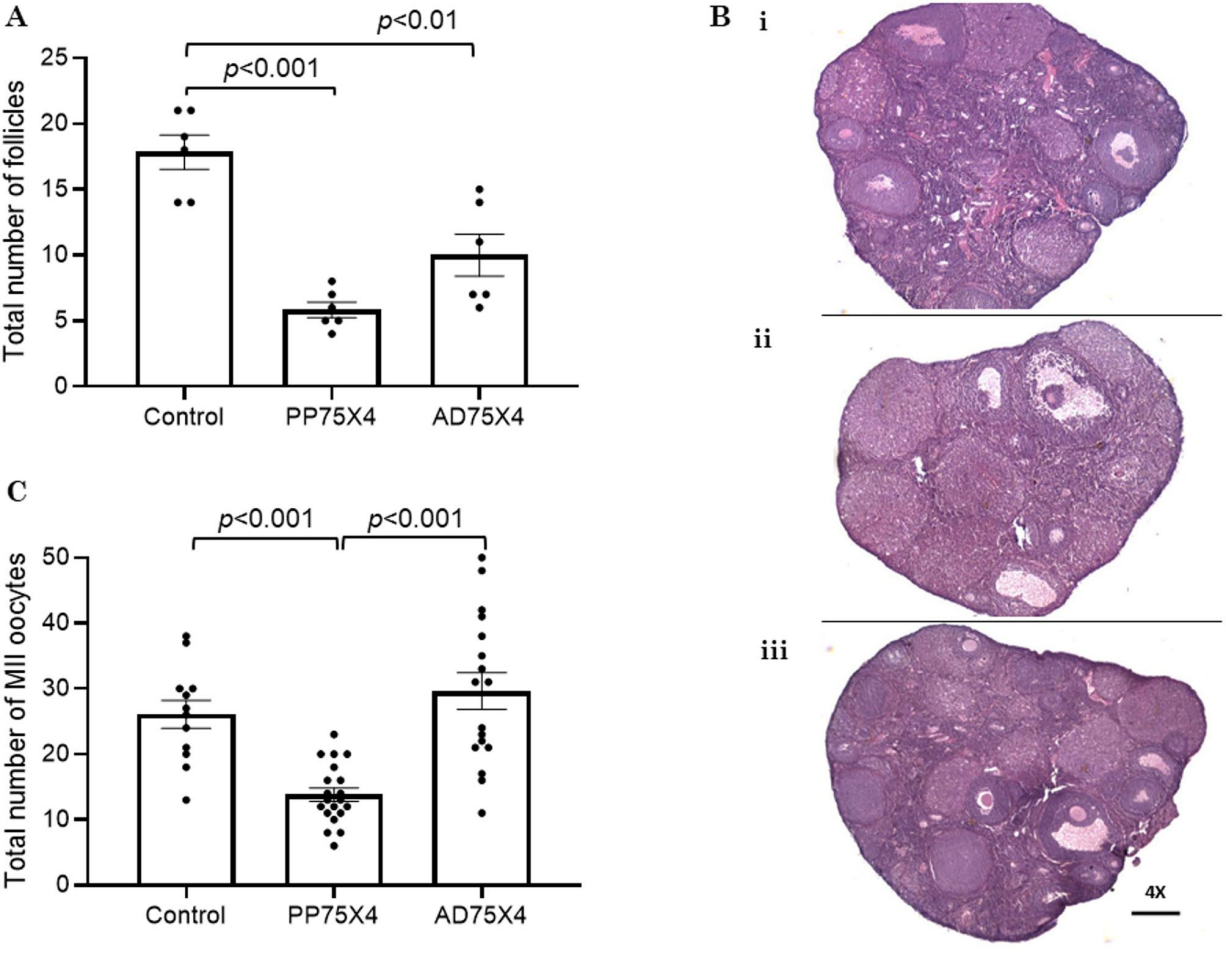
Effect of small multiple CY dose exposure on (**A**) total number of follicles of prepubertal (PP75X4; *N* = 6) and adolescent (AD75X4; *N* = 6) murine ovaries per ovarian section compared to control (*N* = 6) at 22 weeks of life. (**Bi-iii**) Representative histological images of (**Bi**) control, (**Bii**) PP75X4, and (**Biii**) AD75X4 ovaries at 4X magnification. (**C**) Total number of MII oocytes prepubertal (PP75X4; *N* = 20) and adolescent (AD75X4; *N* = 17) murine ovaries compared to control (*N* = 12) at 22 weeks of life.

The total number of follicles in PP75X4 showed a three-fold reduction (*p* < 0.01) compared to the corresponding control. However, the differences between PP75X4 and AD75X4 were not statistically significant (Fig. 1A, Supplementary Table S1). Representative images of ovarian histology have been given in Fig. 1Bi-iii. Post-superovulation PP75X4 females yielded a significantly lower number of metaphase II (MII) oocytes compared to the AD75X4 and control females (*p* < 0.001; Fig. 1C).

### Embryo development, blastocyst quality, and inner cell mass (ICM) proliferation in vitro

**Fig. 2:**
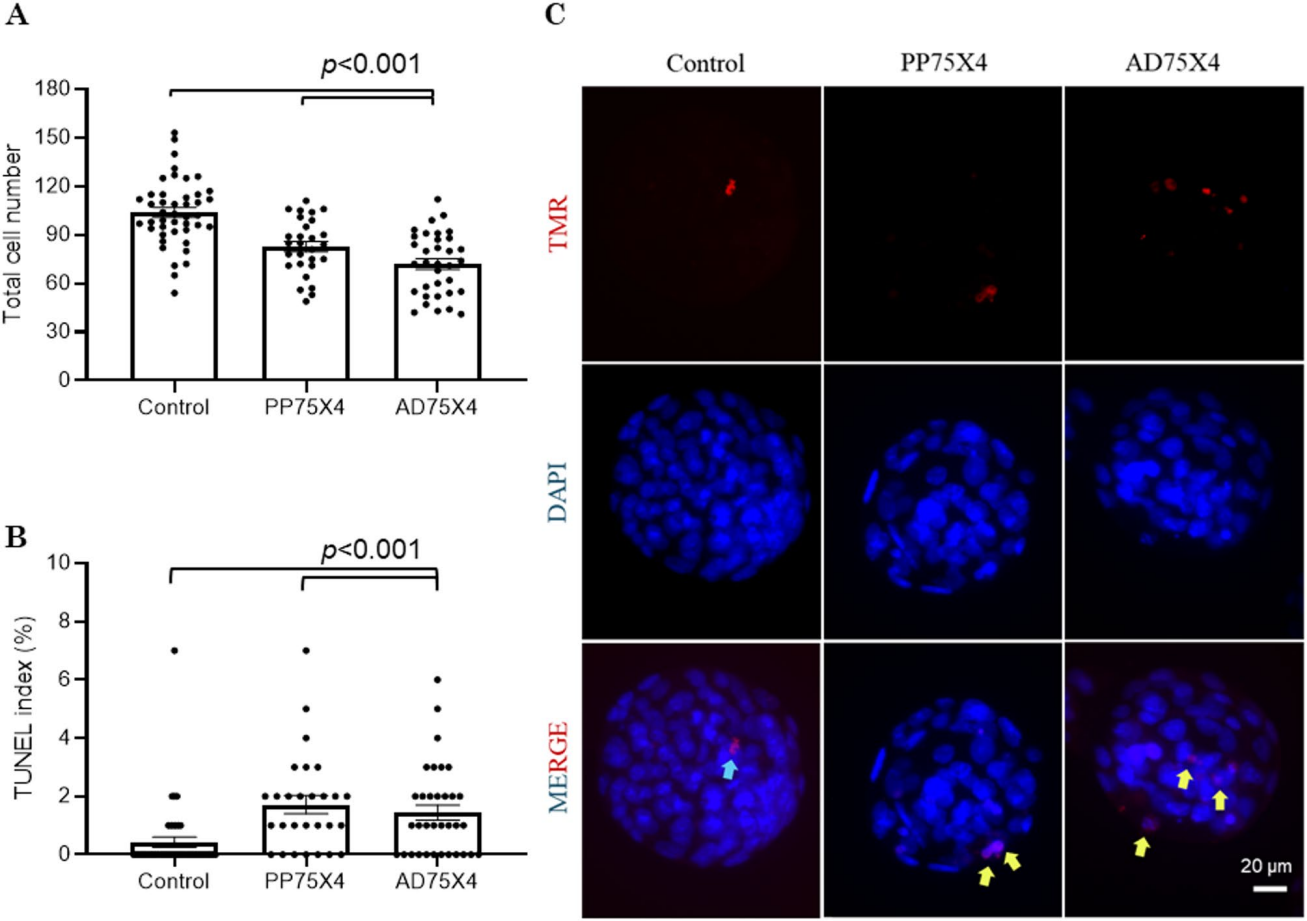
Effect of small multiple CY doses exposure on prepubertal (PP75X4; *N* = 34) and adolescent (AD75X4; *N* = 28) mice blastocysts on (**A**) total cell number and (**B**) apoptotic index in blastocyst obtained in vitro at 120 hpi compared to control (*N* = 44). (**C**) Representative images of the blastocysts showing TUNEL-positive cells (arrow mark).

**Fig. 3:**
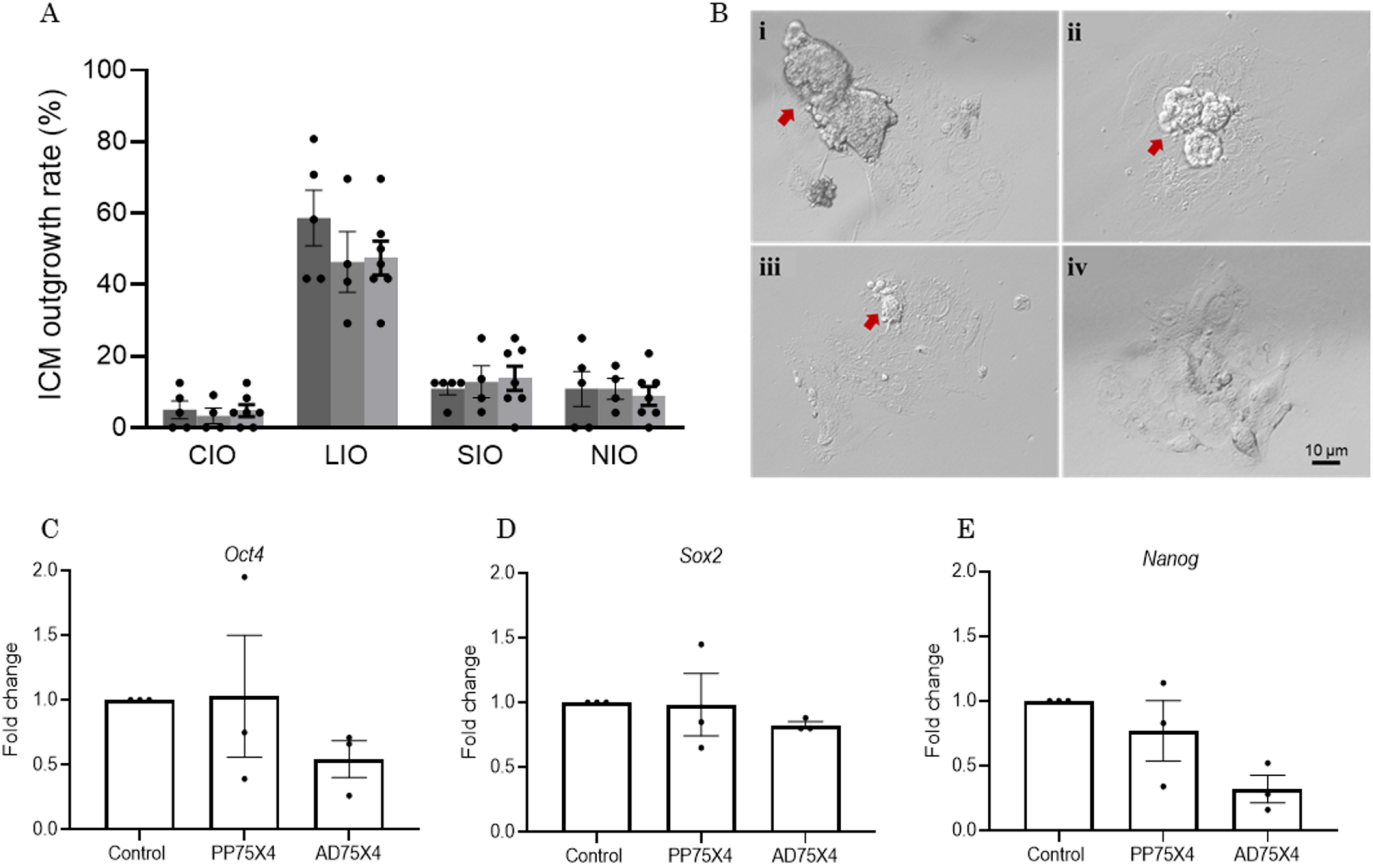
Effect of small multiple CY doses exposure on ICM outgrowth formation at 204 hpi (**A**) control (*N* = 120, dark grey bar), PP75X4 (*N* = 93, grey bar), and AD75X4 (*N* = 167, light grey bar)(**Bi–iv**) Representative images of (**Bi**) completely developed, (**Bii**) large, (**Biii**) small and (**Biv**) no ICM outgrowths. The mRNA level of (**C**) *Oct4*, (**D**) *Sox2*, and (**E**) *Nanog* in ICM outgrowths of control, PP75X4, and AD75X4. The data of three independent trials are represented as mean ± SEM as measured by quantitative real-time PCR using TaqMan chemistry. *Actb* and *gapdh* served as a reference gene, and the expression in the control group was normalized to 1.

Oocytes retrieved from PP75X4 females showed similar fertilization, cleavage, embryo development, and blastocyst formation when compared to the AD75X4 and control females (Table 1). Though TCN of blastocysts was significantly lower (*p* < 0.01; Fig. 2A) and apoptosis was significantly higher (*p* < 0.001; Fig. 2B) in PP75X4 compared to untreated control, the results were comparable with AD75X4 blastocysts. Representative images of blastocysts indicating TUNEL-positive cells have been provided in Fig. 2C. Further, the ability of PP75X4 blastocysts to form ICM outgrowths was comparable with AD75X4 and the untreated control at the end of 96 h extended culture (at 204 hpi) (Fig. 3A). Representative images of ICM outgrowths are in Fig. 3B.

**Table 1:** Effect of small multiple CY doses (75 mg/Kg*4, weekly) on fertilization & pre-implantation embryo development, in vitro.

Group	Number of females (oocytes inseminated)	Fertilization & preimplantation embryo development rate in vitro (% ± SEM)					
		2PN, 2 PB at 10 hpi	2-cell at 24 hpi	4-cell at 48 hpi	Morula at 72 hpi	Blastocyst at 96 hpi	Blastocyst at 120 hpi
Control	12 (313)	264 (84.3 ± 1.9)	241 (91.2 ± 3.1)	203 (75.4 ± 3.0)	221 (82.9 ± 3.5)	211 (78.6 ± 2.6)	217 (80.9 ± 3.1)
PP75X4	20 (277)	219 (77.5 ± 3.2)	192 (86.4 ± 2.4)	148 (66.1 ± 3.7)	179 (80.5 ± 2.9)	155 (69.0 ± 3.4)	161 (72.4 ± 3.3)
AD75X4	17 (504)	357 (70.8 ± 3.0)	325 (90.7 ± 1.9)	248 (72.2 ± 3.0)	289 (81.2 ± 0.9)	253 (71.2 ± 1.3)	275 (78.7 ± 1.6)

### Small multiple dose CY exposure did not affect the expression of pluripotent markers in ICM

The relative expression of *Oct4*, *Sox2*, and *Nanog* mRNA transcripts in ICM outgrowths was comparable between PP75X4 and control. Though the expression level of *Oct4* and *Nanog* was considerably downregulated in the AD75X4 compared to PP75X4 and control, the differences were not statistically significant (Fig. 3C–E).

## Discussion

Small multiple doses of CY resulted in reduced oocyte yield in prepubertal mice. However, the functional competence and quality of embryos derived from such oocytes were comparable between prepubertal and adolescent animals. This observation indicates that the long-term side effects of small multiple CY dose regimens are age independent.

The number and physical integrity of the follicles developed before birth gradually reduce with age until menopause due to physiological atresia^11^. However, gonadotoxic treatments like CY increase follicular activation, leading to premature ovarian failure and early menopause^1^. A recent study revealed that small multiple CY dose regimens (75 mg/kg, four weekly doses) at the prepubertal age showed reduced follicle loss and improved fertility outcomes at the reproductive phase when compared to a single large dose^8^. Therefore, this study compared the effects of small multiple CY dose regimens in prepubertal and adolescent age groups. The histological analyses revealed that prepubertal mice (PP75X4) that received a small multiple CY dose regimen showed a non-significant decline in follicle loss compared to adolescent animals. The susceptibility of prepubertal age groups to CY-induced follicle depletion may be attributed to age-dependent changes in the ovarian microenvironment^12–14^ and could mark their vulnerability to premature aging^15^. Though the follicle depletion was higher in PP75X4 mice in comparison to AD75X4, the differences were not statistically significant. It has been shown that follicular development and oocyte maturation can have enduring negative effects post-CY exposure^9^. Hence, we investigated the oocyte yield and its competence post-CY-exposure, which showed that the PP75X4 group yielded a significantly reduced number of mature oocytes post-superovulation, unlike the AD75X4 and control groups, indicating reduced maturation ability in PP75X4 females.

Earlier, it was shown that CY exposure to prepubertal mice leads to diminished ovarian reserve^9,12,16^. On the other hand, the oocyte quality and its functional competence were unaffected^16^. Similarly, in this study, the fertilizing ability of the oocytes was unaffected in the PP75X4 group, which supported the earlier findings^8,16^. Though embryo development and blastocysts of PP75X4 look morphologically identical to AD75X4 and control, it is noteworthy that embryos may carry DNA damage due to limited checkpoint response during pre-implantation embryo development^17^. This aberrant functional behavior in pre-implantation embryos could result in lowered cell numbers and increased apoptotic index in the blastocysts, as observed in PP75X4 and AD75X4.

Pathways involved in DNA damage repair are activated in implanted embryos and are crucial for normal development^18^. One of the in vitro approaches for assessing the proliferation of the blastocysts is the ICM outgrowth assay that has been effectively incorporated in many studies^16,19–21^. Earlier reports have revealed that reduced proliferation of ICM cells may lead to post-implantation complications, possibly because of the lack of cells forming different lineages^17,22^. However, the ICM outgrowth rates were comparable across all the study groups. This could also be due to the use of only embryos which were progressed to blastocyst for ICM analysis.

The *Oct4*, *Sox2*, and *Nanog* transcripts are crucial for a competent embryo^23^ as they maintain a pluripotent state^24,25^ and regulate germ layer specification post-implantation^26^, whereas their deregulation might affect cell lineages in the post-implantation epiblast and cease development^26,27^. In the current study, the mRNA transcripts *Oct4*, *Sox2*, and *Nanog* in ICM outgrowth cells were comparable between PP75X4 and control. Although AD75X4 demonstrated a reduced level of transcripts, the difference was statistically insignificant. These results indicate that pluripotency is unaffected by the small multiple doses of CY in prepubertal and adolescent mice. Meanwhile, it is noteworthy that the comparable outcomes may be attributed to the assessment being limited to the successfully progressed, completely developed, and large ICM outgrowths. The study is limited by the failure to use of a tumor-bearing mice. Also, evaluating a limited number of sections per ovary may affect the robustness of the follicle count, especially given the inherent variability in ovarian reserve between individual animals. From the clinical perspectives, the use of a single agent, unlike a combination of chemotherapeutic drugs that is used in cancer treatment, is not explored in this study.

## Conclusion

Small multiple doses of CY lead to reduced oocyte yield in prepubertal mice compared to adolescents. However, the functional competence and quality of embryos derived from such oocytes were comparable between prepubertal and adolescent animals, indicating that the long-term side effects of small multiple CY dose regimens are age-independent. This study suggests the comparable functional competence of oocytes between prepubertal and adolescent individuals receiving small multiple doses of CY as part of their treatment.

## Materials and methods

### Animals recruit and CY exposure

All the experiments were performed with ethical approval from the Institutional Animal Ethics Committee (Kasturba Medical College, Manipal & Kasturba Hospital Institutional Ethics Committee, approval #IAEC/KMC/39/2021) and the study was conducted in accordance with the Animal Research: Reporting of In Vivo Experiment (ARRIVE) guidelines. Animal handling, experimentation, and maintenance were done in accordance with the institutional guidelines for animal experimentation. They were housed in the Central Animal Research Facility, in controlled conditions of 23 ± 2 °C, 12 h light-dark cycle, 50 ± 5% humidity, and were fed with standard diet and water *ad libitum*.

This experimental study included a total of seventy-three healthy Swiss albino female mice. One group of females received 4-weekly doses of 75 mg/Kg CY (C7397, Sigma Aldrich, USA) intra-peritoneally (*i.p.*); at postnatal day (Pd) 14, 21, 28, and 35 (*N* = 36; prepubertal exposure and referred to as PP75X4) and the second group at Pd 42, 49, 56 and 63 (*N* = 21; adolescent CY exposure and referred to as AD75X4) (Supplementary Fig. 1). The third group of sixteen females received four weekly doses of normal saline and served as controls for the study.

A minimum of a 12-week recovery period was implemented in the present study as suggested by Meirow et al.^28^. Following the last CY dose that was administered to adolescent females at Pd 63 (week nine), all the females were monitored for fur loss, body weight, and survival till 21 weeks of life. These animals were euthanized by cervical dislocation at 22 weeks of age for assessment of the functional and developmental ability of their oocytes.

## Ovarian histology and follicle count

The ovaries were weighed and fixed in a formalin solution for histological analysis. They were paraffin-embedded and cut into 5 μm sections on slides. Post deparaffinization and rehydration, sections were stained with Hematoxylin and Eosin (H&E), examined under light microscopy (Olympus CX 31, Olympus corporation, Kobe, Japan). Each ovary was analyzed at four levels of 6-micron thickness at 4X, 10X, and 40X magnification. Six ovaries were analysed from each group. The images were obtained using Olympus CX31 and DP23 imaging systems using cellSens Standard software 4.1.1 (build 26344) (Olympus corporation, Kobe, Japan) at 4X. Only the follicles with a clear nucleus of the oocyte were scored, and primordial, primary, and secondary follicles were counted to estimate total follicle count.

### Ovarian stimulation

The cycling female mice at 22 weeks of life were superovulated by injecting 5 IU of pregnant mare serum gonadotropin (PMSG, Sigma Aldrich, St. Louis, MO, USA) intraperitoneally, followed by 10 IU of human chorionic gonadotropin (hCG, Eutrig-HP) 48 h later. Animals were weighed and sacrificed 13 h post-hCG injection to retrieve the Cumulus-Oocyte-Complexes (COCs).

### In vitro fertilization and embryo quality assessment

In vitro fertilization and pre-implantation embryo development in vitro, was performed as explained earlier^16^. Briefly, cauda epididymis from 10–12-week-old Swiss albino male mice (*n* = 10) were retrieved in pre-warmed Earl’s balanced salt solution (EBSS) (Cat. No.: E2888, Sigma Aldrich, St. Louis, MO, USA) supplemented with 0.1% bovine serum albumin (BSA) (Cat. No.: A3311, Sigma Aldrich, St. Louis, MO, USA) and teased to release spermatozoa. The sperm suspension was centrifuged, and the pellet was overlaid with EBSS supplemented with 2.5% BSA and incubated for swim-up for 45 min at 37 °C. The supernatant containing the motile spermatozoa was then collected, inseminated in a 35 mm culture dish (Cat. No.:960010, Tarson, India), and overlaid with oil. Then the superovulated female mice were sacrificed to collect oviducts, and the COCs from the oviducts were released into pre-warmed EBSS supplemented with 0.1% BSA. Oocytes were fertilized in vitro, by incubating with capacitated spermatozoa at 37 °C in 5% CO_2_. At 10 h post insemination (hpi), the oocytes were washed in home-made Potassium simplex optimization medium (KSOM with 0.1% BSA) droplets and observed under an inverted microscope (IX73, Olympus, Japan) to assess fertilization rate. Normally fertilized (having 2 pronuclei and 2 polar bodies) oocytes were cultured in KSOM AA medium and were observed at every 24 h interval for preimplantation embryo progression.

Expanded blastocysts at 108 hpi were fixed in 4% paraformaldehyde for 1 h and Terminal deoxynucleotidyl transferase (TdT) dUTP Nick End Labelling (TUNEL) assay was performed to assess genetic integrity as described earlier^29^, using TUNEL kit by Roche (12156792910, Roche Diagnostics, USA). Stained blastocysts were observed under a fluorescence microscope (Imager-A1; Zeiss, Gottingen, Germany) and counted for 4′,6-diamidino-2-phenylindole (DAPI) stained nuclei (blue fluorescence) as cells for representing total cell number, whereas the TMR-tagged red-fluorescence as apoptotic cells. The apoptotic index was calculated as the percentage of TUNEL-positive cells per blastocyst.

### Assessment of ICM proliferation in vitro

Screening of blastocyst quality by a non-invasive ICM outgrowth assay is considered acceptable to understand their proliferation ability in vitro^20^. The expanded blastocysts at 108 hpi were transferred to pre-coated gelatin plates of multi-well dishes (Cat. No.: D7039, Sigma Aldrich, USA), containing 500 µL Dulbecco’s Modified Eagle Medium (DMEM) 20% supplemented with fetal calf serum (FCS). Following the extended culture of 96 h (at 204 hpi), the proliferation of inner cell mass and trophectoderm was monitored under an inverted phase contrast microscope (IX73, Olympus, Japan), and graded as completely developed ICM outgrowth (CIO), large ICM outgrowth (LIO), small ICM outgrowth (SIO), and no ICM outgrowths (NIO) based on the morphology and size of the outgrowths.

Total RNA was extracted from a minimum of 10 ICM outgrowths (CIO and LIO) using RNAqueous micro kit (Cat.No.: AM1931, Thermo Fisher Scientific, USA) according to the manufacturer’s instruction. cDNA was synthesized using a High-Capacity cDNA RT Kit with RNase Inhibitor (Cat.No.:4374966, Applied Biosystems, Thermo Fisher Scientific, USA), as explained earlier (Uppangala et al., 2021). Using the Taqman premix gene expression assay kit and StepOneTM Real-Time PCR System, a quantitative polymerase chain reaction (qPCR) was performed (Thermo Fisher Scientific, USA). The TaqMan assay (Thermo Fisher Scientific, USA) was employed to evaluate the expression of pluripotency markers *Oct4* (Mm0305391- 7Ig1), *Sox2* (Mm0305- 3810Is1), and *Nanog* (Mm020195- 50Is1). The qPCR outcomes were normalized using *Actb* (Mm00607- 939Is1) and *Gapdh* (Mm99999915_g1) as the reference genes. The qPCR results were analyzed by the delta–delta Ct method. The relative expression of transcripts in the control group was normalized to one.

### Statistical analysis

Data was analyzed using one way analysis of variance (ANOVA) or by Kruskal-Wallis test depending on data distribution, followed by Dunn’s post-hoc test. Categorical data were analyzed using the Chi-square test. Data represented as mean ± SEM. All the tests were performed using the GraphPad InStat 3.0 statistical package (GraphPad Inc., USA), and all the graphs were plotted using GraphPad prism 8.2.1 (GraphPad Software, San Diego, CA, USA). The level of significance was set at 1% throughout the study.

## Supplementary Information

Below is the link to the electronic supplementary material.

Supplementary Material 1

Supplementary Material 2

## Data Availability

All data generated during this study will be provided by the corresponding author upon reasonable request.
