# Supplementary material for: Oocyte and embryo developmental competence following small multiple cyclophosphamide dose administrations in prepubertal female mice are comparable to adolescents
Source: Sci Rep. 2025 Dec 29;16:3460. doi: 10.1038/s41598-025-33355-2 (PMC12834985; doi:10.1038/s41598-025-33355-2)
Supplement: Supplementary file 1 — Supplementary Material 1 [file 41598_2025_33355_MOESM1_ESM.docx]

Supplementary Table S1: Effect of small multiple of CY doses (75 mg/Kg*4, weekly) on ovarian follicular pool

|  | **Control** | | | |  |
| --- | --- | --- | --- | --- | --- |
|  | Primordial follicles | Primary follicles | Secondary follicles | Total follicles |  |
|  | 4 | 3 | 14 | 21 |  |
|  | 3 | 8 | 10 | 21 |  |
|  | 7 | 4 | 8 | 19 |  |
|  | 5 | 5 | 8 | 18 |  |
|  | 6 | 4 | 4 | 14 |  |
|  | | 6 | 5 | 3 | 14 |
| **Mean ± SEM** | **5.2 ± 0.6** | **4.8 ± 0.7** | **7.8 ± 1.6** | **17.8 ± 1.3** |  |

|  | **PP75X4** | | | |
| --- | --- | --- | --- | --- |
|  | Primordial follicles | Primary  follicles | Secondary follicles | Total follicles |
|  | 1 | 1 | 2 | 4 |
|  | 0 | 2 | 3 | 5 |
|  | 0 | 2 | 5 | 7 |
|  | 5 | 1 | 2 | 8 |
|  | 3 | 1 | 1 | 5 |
|  | 4 | 0 | 2 | 6 |
| **Mean ± SEM** | **2.2 ± 0.9** | **1.2 ± 0.3^a^** | **2.5 ± 0.5^a^** | **5.8 ± 0.6^b^** |

|  | **AD75X4** | | | |
| --- | --- | --- | --- | --- |
|  | Primordial follicles | Primary  follicles | Secondary follicles | Total follicles |
|  | 5 | 6 | 4 | 15 |
|  | 4 | 4 | 3 | 11 |
|  | 4 | 5 | 5 | 14 |
|  | 5 | 1 | 1 | 7 |
|  | 3 | 1 | 2 | 6 |
|  | 4 | 1 | 2 | 7 |
| **Mean ± SEM** | **4.2 ± 0.3** | **3.0 ± 0.9** | **2.8 ± 0.6** | **10.0 ± 1.6^a^** |

**^a^**p < 0.01, **^b^**p < 0.001 *vs.* control.
