# Supplementary figures and images for: Oocyte and embryo developmental competence following small multiple cyclophosphamide dose administrations in prepubertal female mice are comparable to adolescents
Source: Sci Rep. 2025 Dec 29;16:3460. doi: 10.1038/s41598-025-33355-2 (PMC12834985; doi:10.1038/s41598-025-33355-2)

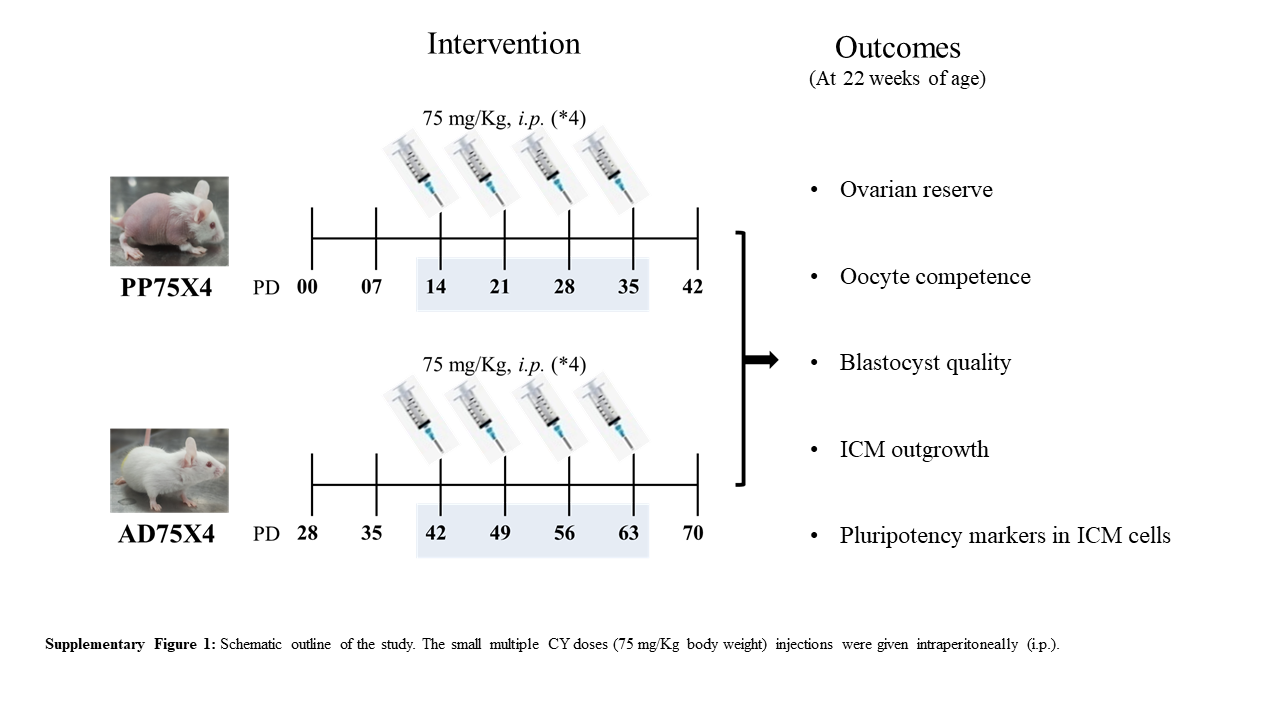

Supplement: Supplementary file 2 — Supplementary Material 2 [file 41598_2025_33355_MOESM2_ESM.tif]
